# The usage and costs of national drug price-negotiated anticancer medicines in a first-tier city in Northeast China: a study based on health insurance data

**DOI:** 10.1186/s12889-024-18820-3

**Published:** 2024-05-14

**Authors:** Bao-Xin Li, Ya-Qun Wang, Yuan-Yuan Yi, Na Zhou, Zi-Xuan Lv, Rui Ma, Xin Li, Ni Yuan

**Affiliations:** 1 School of Public Health, Dalian Medical University, Dalian, China; 2 Department of Respiratory Medicine, First Affiliated Hospital of Dalian Medical University, Dalian, China; 3 Department of Health Policy and Management, Peking University, Beijing, China; 4 School of Pharmacy, Nanjing Medical University, Nanjing, China

**Keywords:** National drug price negotiation, Anticancer medicines, Health insurance

## Abstract

**Background:**

The National Drug Price Negotiation (NDPN) policy has entered a normalisation stage, aiming to alleviate, to some extent, the disease-related and economic burdens experienced by cancer patients. This study analysed the use and subsequent burden of anticancer medicines among cancer patients in a first-tier city in northeast China.

**Methods:**

We assessed the usage of 64 negotiated anticancer medicines using the data on the actual drug deployment situation, the frequency of medical insurance claims and actual medication costs. The affordability of these medicines was measured using the catastrophic health expenditure (CHE) incidence and intensity of occurrence. Finally, we used the defined daily doses (DDDs) and defined daily doses cost (DDDc) as indicators to evaluate the actual use of these medicines in the region.

**Results:**

During the study period, 63 of the 64 medicines were readily available. From the perspective of drug usage, the frequency of medical insurance claims for negotiated anticancer medicines and medication costs showed an increasing trend from 2018 to 2021. Cancer patients typically sought medical treatment at tertiary hospitals and purchased medicines at community pharmacies. The overall quantity and cost of medications for patients covered by the Urban Employee Basic Medical Insurance (UEBMI) were five times higher than those covered by the Urban and Rural Resident Medical Insurance (URRMI). The frequency of medical insurance claims and medication costs were highest for lung and breast cancer patients. Furthermore, from 2018 to 2021, CHE incidence showed a decreasing trend (2.85–1.60%) under urban patients’ payment capability level, but an increasing trend (11.94%–18.42) under rural patients’ payment capability level. The average occurrence intensities for urban (0.55–1.26 times) and rural (1.27–1.74 times) patients showed an increasing trend. From the perspective of drug utilisation, the overall DDD of negotiated anticancer medicines showed an increasing trend, while the DDDc exhibited a decreasing trend.

**Conclusion:**

This study demonstrates that access to drugs for urban cancer patients has improved. However, patients’ medical behaviours are affected by some factors such as hospital level and type of medical insurance. In the future, the Chinese Department of Health Insurance Management should further improve its work in promoting the fairness of medical resource distribution and strengthen its supervision of the nation’s health insurance funds.

**Supplementary Information:**

The online version contains supplementary material available at 10.1186/s12889-024-18820-3.

## Introduction

Cancer is the leading cause of death for the majority of the population in many countries and is a significant obstacle to increasing life expectancy [1]. In December 2020, the GLOBOCAN 2020 database published estimates for the incidence and mortality rates of 36 categories of cancer in 185 countries/regions worldwide. The data revealed that there were 19,292,789 new cancer cases globally and 9,958,133 cancer-related deaths in 2020. Asia, which constitutes 59.5% of the global population, accounts for 49.3% of new cases and 58.3% of cancer deaths [2]. In 2020, China reported 4,568,754 new cancer cases and 3,002,899 cancer-related deaths, representing approximately 23.7% and 30.2% of the global cancer incidence and mortality rates, respectively, for that year [3].

Medicines play a crucial role as essential safeguards and critical approaches in improving the effectiveness of cancer prevention and treatment. In recent years, there has been notably rapid growth in targeted anticancer medicines, such as monoclonal antibodies, in clinical applications due to their significant efficacy. However, as these are predominantly innovative medicines, their high prices deter most patients (even patients in developed countries) from using them [4]. A descriptive study of the use of innovative anticancer drugs from 2000 to 2015 showed that 12% of breast cancer patients in the United States, 27–54% of breast cancer patients in Europe, and 27–49% of breast cancer patients in China had not never used innovative anticancer drugs [5]. Moreover, an empirical study in the United States discovered the higher out-of-pocket costs were correlated with higher rates of the abandonment of oral prescription across cancer patients [6]. Benjamin et al. analyzed the impacts of the healthcare payment system in France and USA on the use of oral anticancer drugs and found that funding of drug reimbursement systems is a critical factor influencing the choice of cancer treatments [7]. In France, although access to innovative anticancer drugs seemed to be relatively equitable, social deprivation was also associated with poorer access [8]. Therefore, lowering medicine cost as a part of a universal health coverage can contribute to improve equal patient access to innovative anticancer drugs. Meanwhile, global spending on anticancer medicines is estimated to increase from US$96 billion in 2016 to US$164 billion in 2020, with projections indicating an increase to US$269 billion by 2025 [9].

A cross-sectional study based on 2012–2014 data revealed that the annual treatment cost for cancer patients in China reached US$9,739, exceeding the average annual household income of US$8,607 during that period [10]. Further research indicates that the exorbitant prices of anticancer medicines impose a significant economic burden on Chinese patients [11, 12].

The National Drug Price Negotiation (NDPN) policy is a significant innovative measure recently implemented for medicines added to the National Reimbursement Drug List (NRDL). This policy aims to meet patients’ medication needs for major, severe and chronic illnesses as well as to improve the accessibility and affordability of innovative medicines [13]. The negotiation process involves the addition of anticancer medicines to the NRDL, thereby benefiting patients by reducing their economic burdens and facilitating innovations in pharmaceutical companies [14]. To better ensure the supply of medicines, medicines included in the NRDL are supplied in most provinces through a ‘dual-channel’ system. This system refers to the mechanism by which negotiated medicines are supplied and clinically used through designated medical institutions and community pharmacies and are concurrently included in the medical insurance payment system [15].

**Fig. 1:**
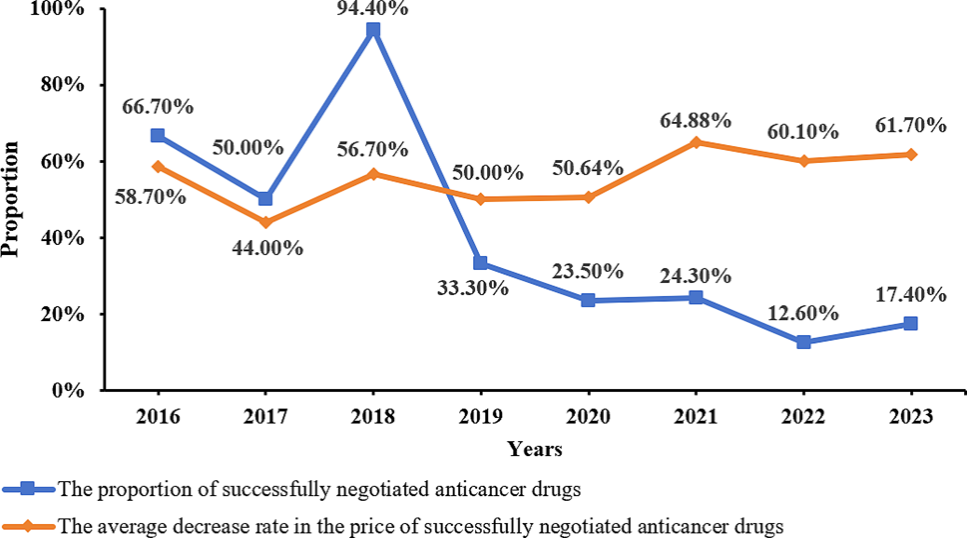
The proportion and average reduction of prices of negotiated anticancer medicines that were successfully negotiated from 2016 to 2023. *Note* Data were obtained from the official announcement of the National Medical Insurance Administration of China and the National Reimbursement Drug List (NRDL)

Starting in 2016, China formally initiated the NDPN policy. To date, it has completed eight rounds and has become a regular practice in the country. Notably, in 2018, the ‘Special Session for Anticancer Medicines’ included 18 medicines in the negotiation, with 17 reaching successful agreements. As of 2023, a total of 398 NDPN medicines are now included in the NRDL during the agreement period, of which 83 are anticancer medicines. The situation and reduction rates of anticancer medicines admitted through NDPN from 2016 to 2023 are illustrated in Fig. 1. The reform of China’s drug price negotiation policy conforms to the trend of global health reform. It is expected that China’s successful experience in drug price negotiation will provide references for other developed or developing countries.

The inclusion of anticancer medicines in the NRDL and the resulting substantial price reductions theoretically alleviate the economic burden on Chinese cancer patients. However, whether the accessibility of these drugs is truly improved depends largely on how well related policies are implemented. In recent years, several scholars have systematically investigated the accessibility of NDPN anticancer medicines. For example, Jiang [16] conducted a study on the accessibility of anticancer medicines and found that the NDPN policy increased the affordability and accessibility of these medicines for patients. Other scholars, such as Huang [17], Liu [18], Zhang [19], Liu [20], Zhu [21], Sun [22], Cai [23], Fang [24], Diao [25], and Ding [26], have investigated the use of NDPN anticancer medicines by utilising hospital procurement data and employing interrupted time series methods. Their results indicated that the implementation of the NDPN policy resulted in a noticeable increase in the use of anticancer medicines, accompanied by a further decrease in drug costs. At the same time, these studies identified substantial disparities in the deployment and use of negotiated anticancer medicines. Issues included variations across different regions, disparities among hospitals within the same region and significant differences between health insurance schemes. However, only hospital procurement data were employed in these studies, and as such, patients’ information was not examined. In other words, these studies were unable to analyse deeply the different effects of NDPN policy on cancer patients with different socioeconomic backgrounds. Moreover, these studies did not investigate the use of negotiated anticancer medicines in community pharmacies under China’s dual-channel policy.

To address this research gap, the present study used health insurance claims data for negotiated anticancer medicines in a first-tier city in northeast China from 2018 to 2021. The analysis focused on changes in the frequency of medical insurance claims and medication costs for cancer patients in the city following the implementation of the NDPN policy. Simultaneously, this study aimed to explore the challenges associated with the local implementation of the policy regarding negotiated anticancer medicines and to provide policy recommendations. The strengths of this study lie in its cross-analysis of the levels of medical institutions and categories of medical treatment, thus offering a comprehensive reflection of changes in patients’ treatment-related behaviours under the NDPN policy.

## Methods

### Data source

The data for this study were sourced from the city’s healthcare security administration. This city is at the forefront of economic development and medical standards in the northeastern region of China, with a resident population of 6.087 million (registered population). The city has 3.662 million urban employees covered by insurance and an additional 2.711 million urban and rural residents covered by the system. Furthermore, the city boasts 140 general and specialised hospitals at or above the secondary level.

**Fig. 2:**
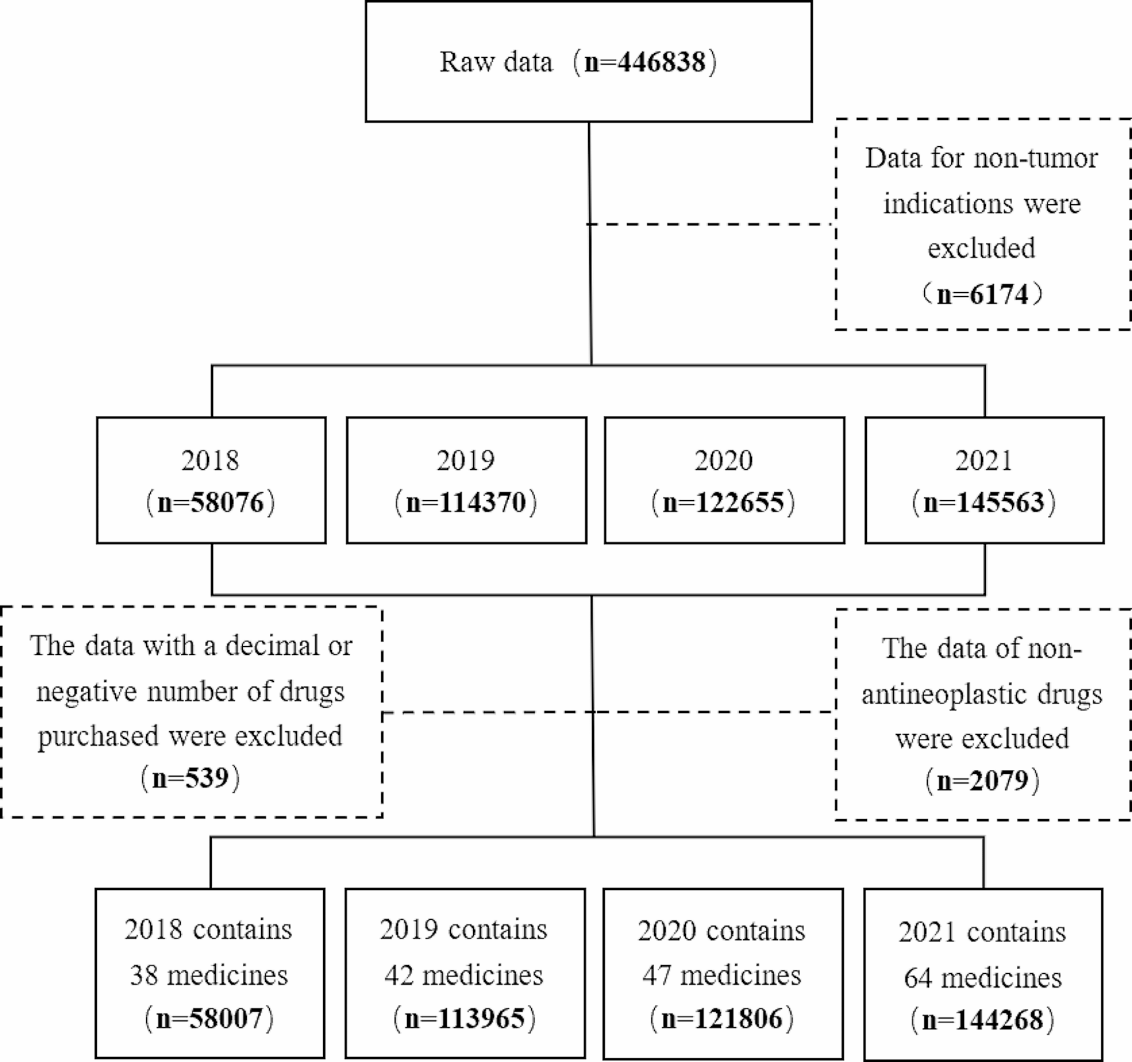
Data filtering process diagram

The author extracted information on negotiated anticancer medicines included in the NRDL from 2018 to 2021. The data used in this study were anonymised and did not contain any information that could be traced back to an individual. This dataset includes patient information and health insurance claims information. Patient information included personal code, person category (e.g. adult or minor), date of birth, and so on. Health insurance claims information encompasses claims time, classifications of medical treatment, names of designated medical institutions, levels of the medical institution, generic names of drugs, dosage and cancer types, among other details. This study was approved by the Ethics Committee of Nanjing Medical University. The original data included some non-anticancer drugs, non-NDPN drugs and illogical drugs (the number of drugs purchased is decimal or negative). After screening, 438,046 records remained. The data filtering process is illustrated in Fig. 2.

### Medicines

From 2016 to 2020, 64 NDPN anticancer medicines were successfully introduced in the city (the numbers of species from 2016 to 2020 were 2, 21, 17, 7, and 17, respectively), comprising 38 oral formulations and 26 injectable formulations [see Additional file 1]. The indications cover a range of cancer types, including lung cancer, breast cancer, colorectal cancer, lymphoma, prostate cancer, gastric cancer and liver cancer, among others.

### Outcome measures

This study analysed the usage and costs of 64 NDPN anticancer medicines in different medical institutions in the city from 2018 to 2021. First, we analysed the usage of 64 NDPN anticancer medicines by frequency of medical insurance claims. Second, we evaluated the medication costs and affordability of 64 NDPN anticancer medicines. Finally, we observed drug utilisation indicators, namely, defined daily doses (DDDs) and defined daily doses cost (DDDc) for a more comprehensive assessment.

We also used the frequency of medical insurance claims to evaluate and measure drug usage. The frequency of medical insurance claims refers to the number of claims submitted by individuals or groups to medical insurance providers within a certain period. When a drug is used frequently, the corresponding frequency of medical insurance claims will be higher; thus, this measure reflects a patient’s demand for drug treatment and the rational use of medicines.

It is common for anticancer medicines to impose significant financial burdens, making them unaffordable for most patients. Therefore, we evaluated the affordability of 64 NDPN anticancer medicines while evaluating medication costs. Several studies have used catastrophic health expenditures (CHE) to assess patients’ medical burden [10, 27]. In the present study, CHE was also used to assess the affordability of medication. The basic idea of CHE is that medical expenditure is considered catastrophic when a family’s out-of-pocket medical expenditure exceeds a certain proportion of its remaining income after meeting the needs of survival [28]. This study used 40% of nonfood expenditure to indicate the ability to pay, because 40% is the threshold recommended by the World Health Organization (WHO) [29]. The average annual household nonfood expenditure was calculated as the annual per capita nonfood expenditure multiplied by the average household size in the current year [30]. The calculation of CHE incidence is based on the number of patients and is defined as follows: Incidence of CHE = Number of patients experiencing CHE/Total number of patients in the sample. The intensity of CHE is measured as the proportion of actual drug expenditure to nonfood consumption expenditure equivalent to the multiple of ability to pay [31].

Furthermore, this study used drug utilisation indicators to analyse changes in the dosage and price of negotiated anticancer medicines. The DDD is the assumed average maintenance dose per day for a drug used for its main indication in adults. We used DDD to standardise medication quantities using DDD data from package inserts. Medication quantities were assessed using DDDs (DDDs = total sales volume/DDD). DDDs represent the number of DDD consumed within a specified time frame; a higher DDD value indicates greater frequency of medication use and a stronger clinical preference for the drug. In addition, DDDs possess the feature of additivity, enabling the comparison of usage frequencies between different drug classes and stages of drug use [32]. Correspondingly, the author employed DDDc (DDDc = total medication costs/DDDs) to evaluate changes in medication costs, in which a higher value indicates higher daily expenditure. Thus, for two medicines with the same indications and efficacy, the disparity in DDDc reflects their economic competitiveness.

To observe drug pricing data over time, along with the market structure features, we explored the impacts of market competition on the usage and costs of representative negotiated anticancer medicines. The small-molecule targeted drugs for the treatment of non-small cell lung cancer (NSCLC) in this study were regarded as within-class competition drugs. The quantities and changes of the DDDs and DDDc of nine small-molecule targeted drugs for non-small-cell lung cancer were observed during the study period.

### Statistical analysis

The author employed descriptive statistical analysis to examine the utilisation of NDPN anticancer medicines in the city from 2018 to 2021. Subsequently, chi-square and Kruskal–Wallis tests were used to explore whether there were differences in the usage of NDPN anticancer medicines among patients in terms of different classifications of medical treatment, levels of medical institution and health insurance schemes. Statistical analyses were performed using SPSS 26.

## Results

### Patient characteristics

The patients’ ages range from 51 to 80 years, and over 80% of the insured patients are covered by the Urban Employee Basic Medical Insurance (UEBMI). Due to the imperfection of the original data, the ‘cancer types’ of some data were missing, which resulted in 41% of the patients with no record of distinguishable cancer type. Subsequent cancer-type studies were conducted with the use of diseases that can be distinguished. Nevertheless, the existing data align closely with the rankings of incidence rates for different cancer categories. Among the cancer types that can be distinguished, lung cancer accounts for the highest proportion at 46.6%, followed by colorectal cancer (13.5%), breast cancer (8.8%), prostate cancer (5.9%) and others. Table 1 presents further details.

**Table 1:** Characteristics of patients included in the analysis

Characteristics	no. (%) of patients (*n* = 44,211)
Age (years)	
0–10	37 (0.1%)
11–20	27 (0.1%)
21–30	218 (0.5%)
31–40	1746 (3.9%)
41–50	4039 (9.1%)
51–60	8983 (20.3%)
61–70	14,004 (31.7%)
71–80	10,322 (23.3%)
81–90	4331 (9.8%)
> 90	504 (1.1%)
Health insurance scheme	
UEBMI	35,950 (81.3%)
URRMI	8261 (18.7%)
Cancer type	
Diseases that can be distinguished	25,969 (58.7%)
Lung cancer	12,092 (46.6%)
Colorectal cancer	3500 (13.5%)
Breast cancer	2283 (8.8%)
Prostate cancer	1531 (5.9%)
Gastric cancer	1216 (4.7%)
Lymphoma	1125 (4.3%)
Liver cancer	735 (2.8%)
Other cancer	3487 (13.4%)
Diseases that can’t be distinguished	18,242 (41.3%)

*UEBMI: the Urban Employee Basic Medical Insurance; URRMI: the Urban and Rural Resident Medical Insurance; Cancer type could not be distinguished in 41% of patients

### Differences in the frequency of medical insurance claims and medication costs

Frequency of medical insurance claims perspective: Vertically, patients primarily settle their accounts in tertiary hospitals (73%), followed by community pharmacies (26.2%), with secondary and primary hospitals accounting for the most minor proportion (< 1%). Horizontally, patients predominantly settle their accounts through inpatient specialty drugs (62.1%), followed by outpatient (29.6%) and outpatient major diseases or chronic diseases (6.5%). The least frequent reimbursement occurs through regular outpatient visits (1.8%). The frequency of medical insurance claims for UEBMI beneficiaries is nearly 5 times that of Urban and Rural Resident Medical Insurance (URRMI) beneficiaries. From 2018 to 2021, there is an increasing trend in the frequency of medical insurance claims, with the most significant increase observed from 2018 to 2019 (from 13.2 to 26.0%). The frequency of medical insurance claims for injectable formulations is more than 5 times that of oral formulations. In terms of cancer type, lung cancer has the highest settlement frequency (32.1%), followed by breast cancer (7%) and colorectal cancer (5.5%), among others (see Table 2 for details). Furthermore, based on the chi-square test results, patients with different categories of medical treatment, health insurance schemes, settlement years, dosage forms and cancer types show statistically significant differences in the frequency of medical insurance claims across different levels of medical institutions.

**Table 2:** Frequency of medical insurance claims for different categories of patients in designated medical institutions in the city and the corresponding proportions

GROUP	Medical institutions level			Frequency of medical insurance claims	*P*
	Primary and secondary hospitals	Tertiary hospitals	Community pharmacies		
**Frequency of medical insurance claims**	2765 (0.6%)	320,377 (73.1%)	114,904 (26.2%)	438,046 (100%)	*P* < 0.001
**Categories of medical treatment**					*P* < 0.001
Outpatient	319 (0.1%)	5274 (1.2%)	2262 (0.5%)	7855 (1.8%)	
Outpatient major diseases or chronic diseases	111 (0.0%)	25,737 (5.9%)	2782 (0.6%)	28,630 (6.5%)	
Outpatient specialty drugs	3 (0.0%)	19,865 (4.5%)	109,860 (25.1%)	129,728 (29.6%)	
Inpatient	2332 (0.5%)	269,501 (61.5%)	0 (0)	271,833 (62.1%)	
**Health insurance schemes**					*P* < 0.001
URRMI	697 (0.2%)	59,191 (13.5%)	14,972 (3.4%)	74,860 (17.1%)	
UEBMI	2068 (0.5%)	261,186 (59.6%)	99,932 (22.8%)	363,186 (82.9%)	
**Years**					*P* < 0.001
2018	214 (0.0%)	47,429 (10.8%)	10,364 (2.4%)	58,007 (13.2%)	
2019	449 (0.1%)	87,825 (20.0%)	25,691 (5.9%)	113,965 (26.0%)	
2020	907 (0.2%)	95,711 (21.8%)	25,188 (5.8%)	121,806 (27.8%)	
2021	1195 (0.3%)	89,412 (20.4%)	53,661 (12.3%)	144,268 (32.9%)	
**Dosage form**					*P* < 0.001
Oral medicine	215 (0.0%)	17,038 (3.9%)	50,127 (11.4%)	67,380 (15.4%)	
Injection medicine	2550 (0.6%)	303,339 (69.2%)	64,777 (14.8%)	370,666 (84.6%)	
**Cancer type**					*P* < 0.001
Lung cancer	293 (0.0%)	118,715 (27.1%)	21,564 (4.9%)	140,572 (32.1%)	
Colorectal cancer	154 (0.0%)	19,847 (4.5%)	4144 (0.9%)	24,145 (5.5%)	
Breast cancer	31 (0.0%)	20,167 (4.6%)	10,333 (2.4%)	30,531 (7.0%)	
Prostate cancer	94 (0.0%)	12,394 (2.8%)	3170 (0.7%)	15,658 (3.6%)	
Gastric cancer	56 (0.0%)	8709 (2.0%)	207 (0.0%)	8972 (2.0%)	
Lymphoma	76 (0.0%)	2254 (0.5%)	5783 (1.3%)	8113 (1.9%)	
liver cancer	48 (0.0%)	9716 (2.2%)	934 (0.2%)	10,698 (2.4%)	
Other cancer	338 (0.1%)	25,571 (5.8%)	4748 (1.1%)	30,657 (7.0%)	
Diseases can’t be distinguished	1644 (0.4%)	103,004 (23.5%)	64,052 (14.6%)	168,700 (38.5%)	

*UEBMI: the Urban Employee Basic Medical Insurance; URRMI: the Urban and Rural Resident Medical Insurance

Medication costs perspective: Vertically, patients’ costs primarily originate from community pharmacies (72.4%), followed by tertiary hospitals (27.4%), with costs from secondary and primary hospitals accounting for only 0.5%. Horizontally, most patients’ costs are incurred through outpatient specialty drugs (82.5%), followed by inpatient drugs (12.8%). Outpatient visits and outpatient major diseases or chronic diseases account for nearly 5%. The costs for patients covered by UEBMI are more than 5 times those of patients covered by URRMI. Furthermore, the costs for patients using injectable formulations (59.2%) are higher than those using oral formulations (40.8%) (with 38 oral medicines and 26 injectable medicines in the dataset). From 2018 to 2021, overall costs reveal a rising trend, with significant increases observed from 2018 to 2019 and from 2020 to 2021. In terms of cancer type, lung cancer patients incur the highest costs (22.1%), followed by breast cancer (8.3%), lymphoma (5.1%) and others (see Table 3 for details). According to the results of the Kruskal–Wallis test, there are significant differences in medication costs among different groups in the patient categories.

**Table 3:** Medication costs for different categories of patients in designated medical institutions in the city and the corresponding proportions

GROUP	Medical institutions level			Total medication costs (10,000 CNY)	*P*
	Primary and secondary hospitals	Tertiary hospitals	Community pharmacies		
**Medication costs**	243 (0.2%)	29,144 (27.4%)	77,034 (72.4%)	106,421(100%)	*P* < 0.001
**Categories of medical treatment**					*P* < 0.001
Outpatient	39 (0.0%)	1110 (1.0%)	124 (0.1%)	1273 (1.2%)	
Outpatient major diseases or chronic diseases	19 (0.0%)	3477 (3.3%)	173 (0.2%)	3669 (3.5%)	
Outpatient specialty medicines	2 (0.0%)	11,082 (10.4%)	76,737 (72.1%)	87,821 (82.6%)	
Inpatient	184 (0.2%)	13,475 (12.7%)	0 (0)	13,659 (12.7%)	
**Health insurance schemes**					*P* < 0.001
URRMI	115 (0.1%)	6635 (6.2%)	9277 (8.7%)	16,027 (15.1%)	
UEBMI	129 (0.1%)	22,509 (21.2%)	67,757 (63.7%)	90,395 (84.9%)	
**Years**					*P* < 0.001
2018	10 (0.0%)	2983 (2.8%)	8236 (7.7%)	11,229 (10.6%)	
2019	19 (0.0%)	6706 (6.3%)	19,664 (18.5%)	26,389 (24.8%)	
2020	39 (0.0%)	8876 (8.3%)	16,289 (15.3%)	25,204 (23.7%)	*P* < 0.001
2021	176 (0.2%)	10,578 (9.9%)	32,845 (30.9%)	43,599 (41.0%)	
**Dosage form**					
Oral medicine	25 (0.0%)	7854 (7.4%)	35,552 (33.4%)	43,431 (40.8%)	
Injection medicine	218 (0.2%)	21,290 (20.0%)	41,482 (39.0%)	62,990 (59.2%)	
**Cancer type**					*P* < 0.001
Lung cancer	10 (0.0%)	8935 (8.4%)	14,548 (13.7%)	23,493 (22.1%)	
Colorectal cancer	6 (0.0%)	1490 (1.4%)	2655 (2.5%)	4151 (3.9%)	
Breast cancer	3 (0.0%)	2626 (2.5%)	6223 (5.8%)	8852 (8.3%)	
Prostate cancer	15 (0.0%)	2183 (2.1%)	2791 (2.6%)	4989 (4.7%)	
Gastric cancer	3 (0.0%)	418 (0.4%)	131 (0.1%)	551 (0.5%)	
Lymphoma	17 (0.0%)	473 (0.4%)	4923 (4.6%)	5412 (5.1%)	
liver cancer	2 (0.0%)	313 (0.3%)	706 (0.7%)	1021 (1.0%)	
Other cancer	41 (0.0%)	1234 (1.2%)	4188 (3.9%)	5463 (5.1%)	
Diseases can’t be distinguished	152 (0.1%)	11,494 (10.8%)	40,843 (38.4%)	52,489 (49.3%)	

*UEBMI: the Urban Employee Basic Medical Insurance; URRMI: the Urban and Rural Resident Medical Insurance

### Availability and affordability of medications for common cancer disease

The availability of negotiated anticancer drugs in the city is good. Among 64 drugs approved from 2016 to 2020, only one Chinese patent medicine (Shi Dao Ping San) is listed outside the database. From the basic drug information, it can be observed that the average time intervals from approval to inclusion in the NRDL for NDPN anticancer medicines brought into coverage (excluding medicines not included for the first time) from 2016 to 2020 are 6, 8.7, 4.9, 2.4 and 0.9 years, respectively.

**Fig. 3:**
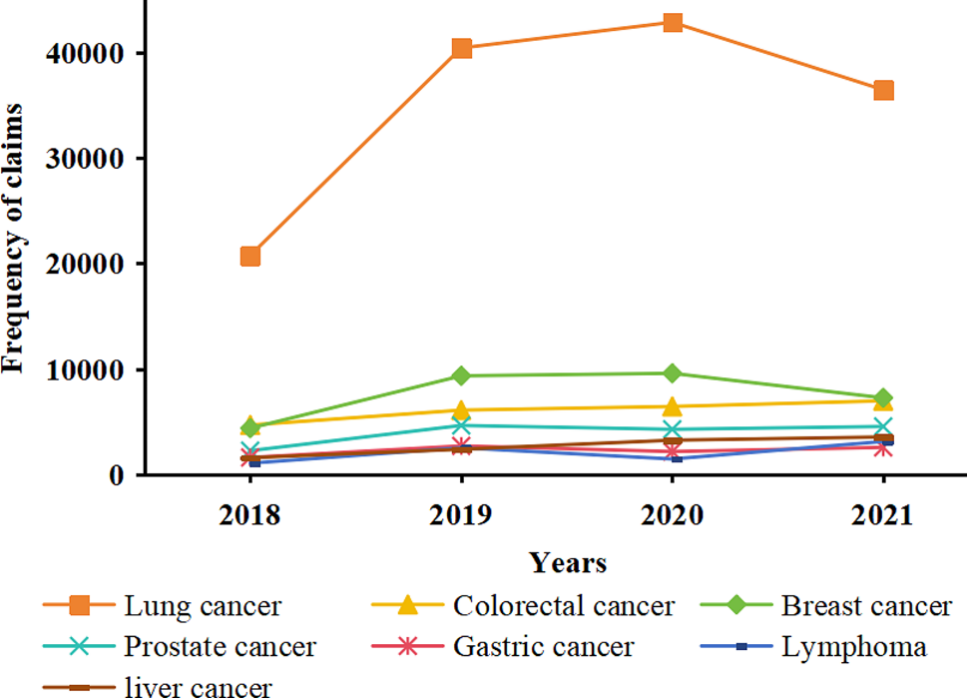
Frequencies of medical insurance claims for common cancer types

**Fig. 4:**
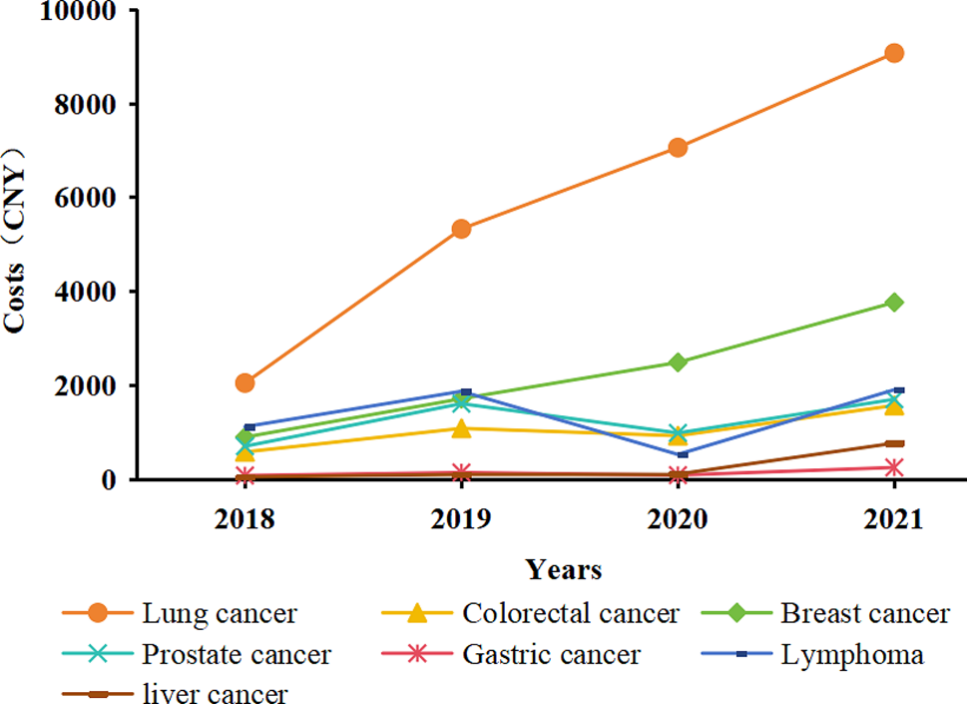
Medication costs for common cancer types

Upon examining the usage and reimbursement trends of negotiated anticancer medications for common cancer categories from 2018 to 2021, the results show that lung cancer and breast cancer exhibit overall upward trajectories in the frequency of medical insurance claims and medication costs. Notably, lung cancer patients consistently demonstrate higher values in their frequency of medical insurance claims and medication costs compared with other cancer types and show a more pronounced growth trend. However, there was a noticeable decline in the frequency of medical insurance claims for lung cancer and breast cancer patients from 2020 to 2021, while medication costs continued to rise. This finding suggests an increase in average per-patient medication costs. In contrast, several other cancer types generally show a stable trend during the study. Figures 3 and 4 present details of these results.

Overall, under rural patients’ payment capability level, the average CHE incidence rate is more than 4 times that of urban patients, while the intensity is nearly twice as much. In comparison, under urban patients’ payment capability levels, the average CHE incidence rate for common cancer categories initially increased, followed by a decrease from 2.85% in 2018 to 1.6% in 2021. The average intensity continuously increased from 0.55 times in 2018 to 1.26 times in 2021. Under rural patients’ payment capability levels, the average CHE incidence rate for common cancer categories shows a fluctuating trend of initial increase, followed by a decrease and then an increase again, increasing from 11.94% in 2018 to 18.42% in 2021. The average intensity also shows an initial increase, followed by a decrease from 1.27 times in 2018 to 1.74 times in 2021. For common cancer categories, whether in urban or rural areas, stomach and liver cancers have the lowest average CHE incidence rates, while lymphoma has the highest. In terms of CHE intensity for common cancer categories, stomach and liver cancers still have the lowest intensity, while prostate cancer has the highest. Specific data can be found in Tables 4 and 5.

**Table 4:** Incidence and intensity of CHE for common cancer types under the condition of urban patients’ payment capability level

Categories of cancer	Incidence of CHE				Intensity of CHE			
	2018	2019	2020	2021	2018	2019	2020	2021
Lung cancer	0.00%	3.17%	4.69%	2.54%	0.00	1.74	1.92	1.45
Colorectal cancer	0.00%	8.01%	1.96%	2.29%	0.00	1.06	1.54	1.46
Breast cancer	0.31%	0.90%	3.57%	3.11%	1.12	1.23	1.23	1.21
Prostate cancer	5.94%	9.15%	2.07%	0.84%	1.45	1.87	1.28	1.37
Gastric cancer	0.00%	0.00%	0.00%	0.29%	0.00	0.00	0.00	1.23
Lymphoma	13.72%	21.84%	4.22%	1.92%	1.29	1.24	1.20	1.10
Liver cancer	0.00%	0.00%	0.00%	0.20%	0.00	0.00	0.00	1.02
Average	2.85%	6.15%	2.36%	1.60%	0.55	1.02	1.02	1.26

**Table 5:** Incidence and intensity of CHE for common cancer type under the condition of rural patients’ payment capability level

Categories of cancer	Incidence of CHE				Intensity of CHE			
	2018	2019	2020	2021	2018	2019	2020	2021
Lung cancer	7.28%	9.87%	10.91%	19.01%	1.44	2.55	2.99	1.85
Colorectal cancer	0.07%	9.84%	6.30%	9.61%	1.06	2.45	2.38	2.10
Breast cancer	8.02%	8.72%	16.05%	37.08%	1.42	1.89	1.93	1.74
Prostate cancer	10.45%	12.30%	4.35%	5.70%	2.90	4.20	2.41	1.82
Gastric cancer	0.00%	0.00%	0.00%	5.75%	0.00	0.00	0.00	1.64
Lymphoma	57.76%	60.30%	35.86%	39.08%	2.09	2.36	1.78	1.60
Liver cancer	0.00%	0.00%	0.00%	12.70%	0.00	0.00	1.25	1.46
Average	11.94%	14.43%	10.50%	18.42%	1.27	1.92	1.82	1.74

### Utilisation of negotiated anticancer medicines

**Fig. 5:**
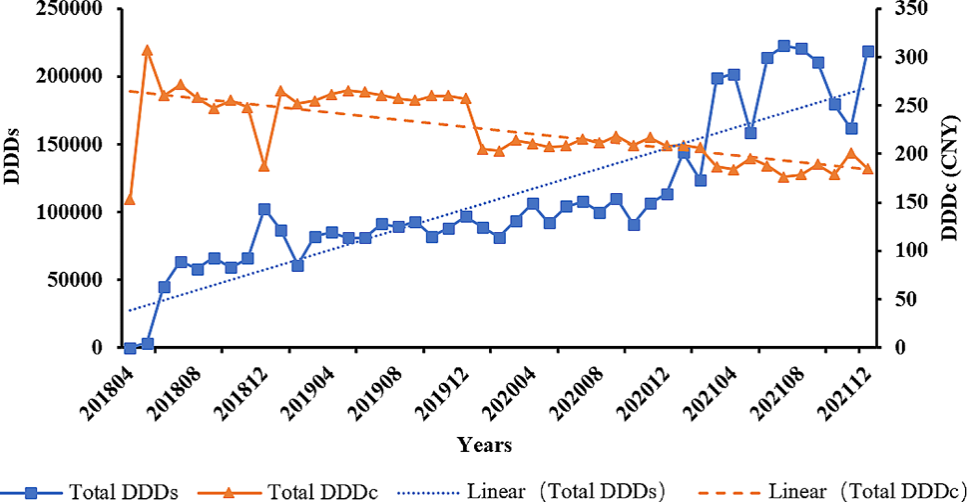
The trends of total DDDs and DDDc for all medicines

The graph shows that the DDDs for these medicines exhibit a continuous upward trend, while the DDDc shows a consistent downward trend. The increase in drug use and the decrease in cost can also indicate that the accessibility of drugs to cancer patients is improving (see Fig. 5 for details).

**Fig. 6:**
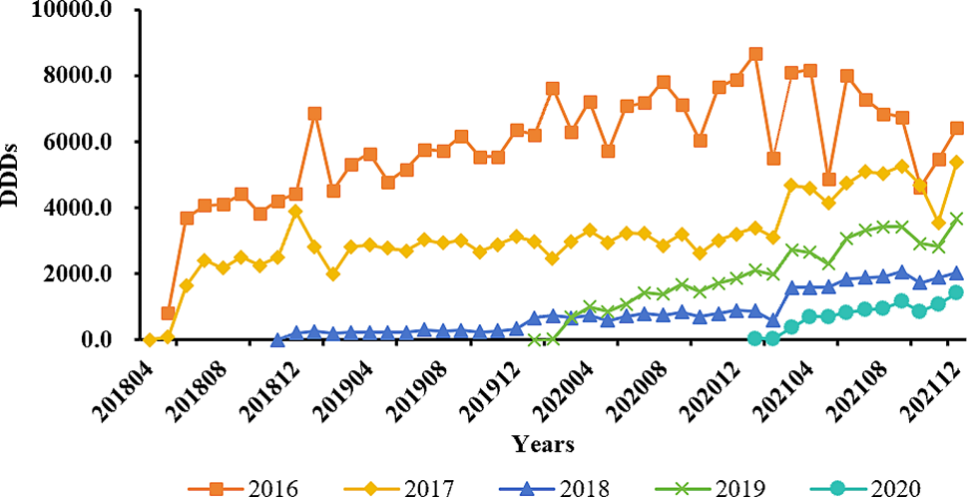
The average DDDs for medicines included each year from 2016 to 2020

**Fig. 7:**
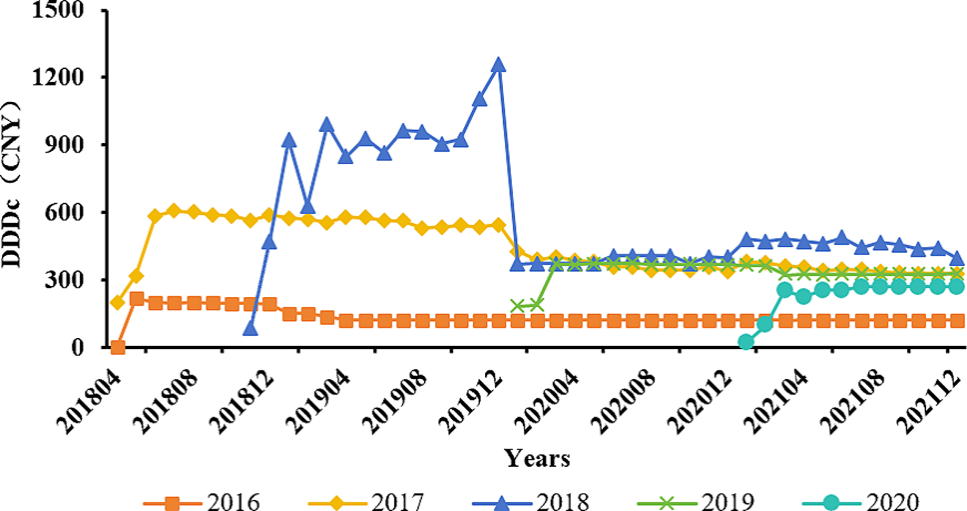
The average DDDc for medicines included each year from 2016 to 2020

As shown in the graphs, in 2016, the drug usage frequency was the highest, and the daily costs were the lowest. In comparison, newly admitted medicines initially exhibited higher daily costs (around 1000 yuan overall) in the early inclusion period in 2018 and throughout 2019. However, starting in 2020, the costs began to decline to around 500 yuan and continued to maintain this level (see Figs. 6 and 7 for details).

### Influence of market factors on the dosage of NDPN anticancer medicines

**Fig. 8:**
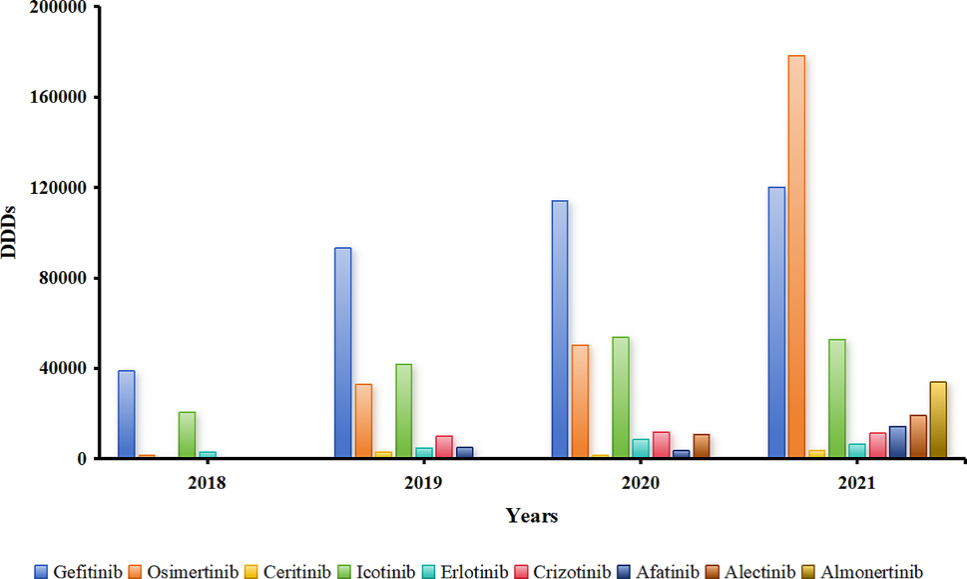
DDDs of small-molecule targeted drugs for the treatment of NSCLC

**Fig. 9:**
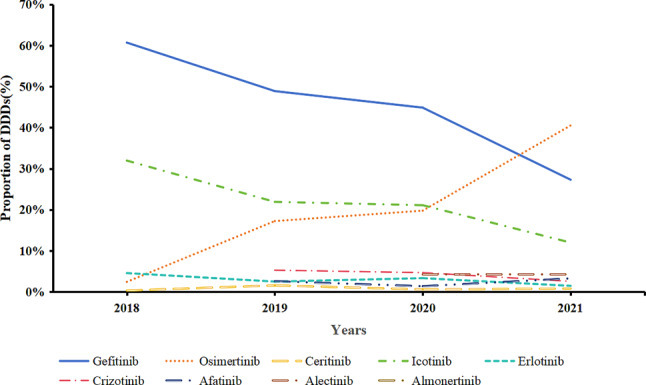
Proportions of DDDs of small-molecule targeted drugs for the treatment of NSCLC

**Fig. 10:**
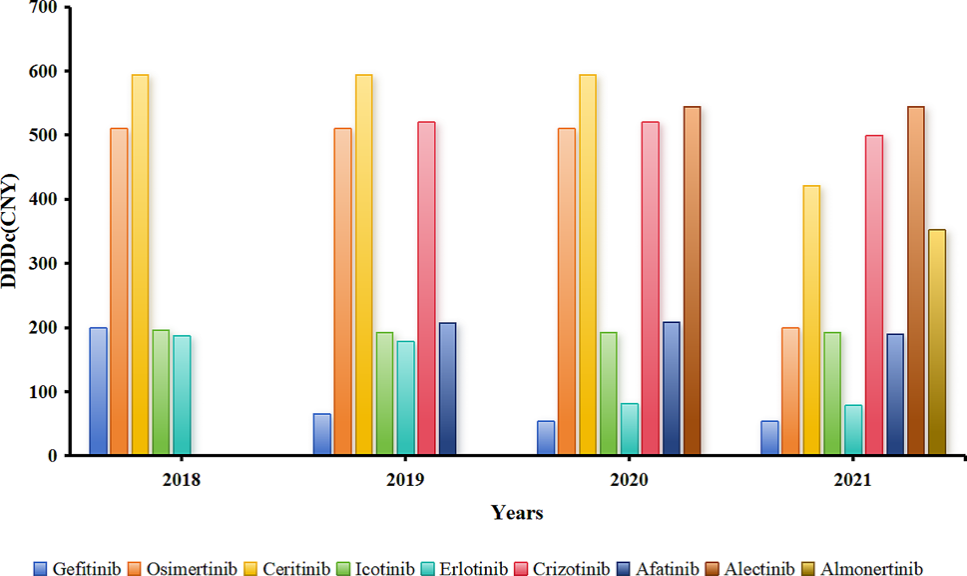
DDDc of small-molecule targeted drugs for the treatment of NSCLC

Gefitinib and Icotinib, which were included in 2016, had the highest DDDs in 2018, 2019, and 2020, as shown in Figs. 8 and 9, respectively. However, with the inclusion of Osimertinib in the medical insurance list in 2018, the proportion of DDDs with Gefitinib and Icotinib gradually decreased. Combined with Figs. 8, 9 and 10, the results show that with the further reduction of the price of Osimertinib, the demand further increased. Meanwhile, in terms of the medical insurance access to drugs, such as Afatinib and Almonertinib, the proportions of DDDs of Gefitinib and Icotinib further decreased.

## Discussion

The findings of this study indicate that the overall accessibility of NDPN anticancer medicines in the city is good; however, there are still some areas of concern that require further attention.

While the majority of the city’s cancer patients tend to seek treatment at tertiary hospitals (73.1%), most reimbursements occur at community pharmacies (72.4%). These patterns could be attributed to the impact of the dual-channel policy, wherein the city has already included most of the negotiated anticancer medicines in the dual-channel drug supply management system. In addition, a uniform payment policy has been implemented. Several studies have shown that the dual-channel policy plays an important role in meeting the medication needs of patients, reducing their economic burdens and improving the quality of medical services [33, 34]. Furthermore, the frequencies of medical insurance claims (0.6%) and medication costs (0.2%) in primary and secondary hospitals are very low, indicating that the cancer diagnosis and treatment capabilities, as well as the drug inventory levels, of primary medical institutions require improvement. With the gradual transformation of cancer diseases into chronic conditions, the comprehensive management of cancer patients in primary medical institutions has become increasingly essential.

Meanwhile, the frequency of medical insurance claims and medication costs in UEBMI-covered patients is approximately 5 times that in URRMI-covered patients. This discrepancy may be attributed to differences in the reimbursement benefits of these medical insurance schemes, a point supported by previous research [28, 35, 36].

The data shows that the cost of negotiated anticancer medicines in 2021 was 3 times higher than in 2018. The potential reasons include the following: Firstly, medical progress and technological innovation have led to the emergence of new drugs, which also entail additional high costs [37]. Secondly, the aging population and the rising incidence of cancer result in an increasing number of patients. One study showed that the elderly population in the United States accounted for about 56% of all cancer cases and 69% of all cancer death [38]. In comparing the frequencies of medical insurance claims and the costs of other diseases, lung cancer (32.1% and 22.1%, respectively) has the highest proportion and the most noticeable growth trend, followed by breast cancer (7.0% and 8.3%, respectively). Related studies have drawn similar conclusions [39–42]. Thirdly, policy and market factors can influence the pricing of new drug [43] and contribute to higher drug costs, such as lower prices of drugs after they are included in medical insurance, leading to a significant increase in demand. Through the analysis of drug utilisation in NSCLC, we found that patients favoured medicines with lower prices, all else being equal. However, the market structure is likely to change as prices for other drugs fall and more drugs become covered. A new generation of drugs has replaced the market share of the old generation of drugs; therefore, the former will be actively withdrawn from the market. Therefore, policymakers should pay close attention to changes in the market for anticancer medicines. On the one hand, they must ensure rapid drug approval so that new drugs with definite efficacy, urgent demand and affordable prices can be quickly marketed and made available to the public. On the other hand, competition is encouraged to further reduce drug prices in the market.

In terms of the incidence and intensity of CHE, the average incidence and intensity of CHE among rural residents are about 4 times and 2 times those of urban residents, respectively, indicating that their economic burdens are heavier. In addition, the incidence and intensity of CHE show a fluctuating trend, which may be due to the approval of new drugs so that patients can receive more effective drugs, as well as the heavier economic burdens compared with the past treatments. As of 2021, breast cancer (3.11%) has the highest incidence of CHE among urban patients, while lymphoma (39.08%) has the highest incidence among rural patients. Thus, it is recommended that policies be implemented in favour of these cancer types, which impose heavier burdens on patients compared with others. In addition, it is necessary to integrate medical assistance policies that focus on helping low-income impoverished groups and improving the expenditure target identification mechanism to prevent patients from falling into poverty or returning to poverty due to their illness [44].

From the point of view of drug utilisation, the overall DDDs of negotiated anticancer medicines show an increasing trend, while DDDc show a decreasing trend. Coincidentally, the findings of a study performed in the US by Gonzales et al. suggested that expansions in health insurance coverage mitigated the effects of growing prescription drug costs to some extent for cancer survivors [45]. Their results demonstrated that there were overall improvements in cancer patient access to innovative drugs, despite increasing prescription drug spending. Our finding also suggests that, overall, the accessibility of medication for patients has been improved to some extent. However, there is a caveat that excessive DDDs may lead to abuse and unnecessary medical costs. In contrast, low DDDs may mean patients’ restrictions on drug use and necessary treatment. Therefore, the frequency of medical insurance claims should be matched with the actual medical needs of patients to avoid the overuse of medicines, while ensuring that patients receive the necessary treatment.

This study has several limitations. First, the research findings are based solely on one city, so they may not be extrapolated to other regions. Second, the data from medical insurance reimbursement records do not include uninsured patients. However, the city’s medical insurance has achieved broad coverage, minimising the potential impact of the exclusion of uninsured patients on the results. Third, the calculation of household nonfood expenditure in this study is based on per capita nonfood expenditure and the average number of people per household, which may lead to discrepancies in the CHE incidence and intensity compared with the actual results. Finally, due to data extraction and database limitations, some data may not be included, and specific data may not be further analysed, resulting in potential deviations between the study results and the actual situation.

## Conclusion

The results showed that the availability of negotiated anticancer medicines for cancer patients in the city is good. However, there is still much room for improvement in terms of the affordability of these medicines, especially for rural patients. The patients’ medical behaviours are mainly affected by the medical institution level and health insurance schemes. Thus, in the future, policymakers should focus on the construction of primary medical institutions and the equitable provision of medical services. In addition, more real-world data are needed to verify the impact of NDPN policies on patients’ access to anticancer drugs in other parts of China.

### Electronic supplementary material

Below is the link to the electronic supplementary material.

Supplementary Material 1

## Data Availability

The data that support the findings of this study are available from Healthcare Security Administrations but restrictions apply to the availability of these data, which were used under license for the current study, and so are not publicly available. Data are however available from the authors upon reasonable request and with permission of Healthcare Security Administrations.
